# The Photoinduced Response of Antimony from Femtoseconds to Minutes

**DOI:** 10.1002/adma.202414687

**Published:** 2025-01-13

**Authors:** Sebastian Walfort, Nils Holle, Julia Vehndel, Daniel T. Yimam, Niklas Vollmar, Bart J. Kooi, Martin Salinga

**Affiliations:** ^1^ Institute of Materials Physics University of Münster Wilhelm‐Klemm‐Str. 10 48149 Münster Germany; ^2^ Zernike Institute for Advanced Materials University of Groningen Nijenborgh 3 Groningen 9747 The Netherlands

**Keywords:** optical memory, phase change material, phase transition, supercooled liquid, ultrafast response

## Abstract

As a phase change material (PCM), antimony exhibits a set of desirable properties that make it an interesting candidate for photonic memory applications. These include a large optical contrast between crystalline and amorphous solid states over a wide wavelength range. Switching between the states is possible on nanosecond timescales by applying short heating pulses. The glass state is reached through melting and rapid quenching through a supercooled liquid regime. While initial and final states are easily characterized, little is known about the optical properties on the path to forming a glass. Here we resolve the entire switching cycle of antimony with femtosecond resolution in stroboscopic optical pump‐probe measurements and combine the experimental results with ab‐initio molecular dynamics simulations. The glass formation process of antimony is revealed to be a complex multi‐step process, where the intermediate transient states exhibit distinct optical properties with even larger contrasts than those observed between crystal and glass. The provided quantitative understanding forms the basis for exploitation in high bandwidth photonic applications.

## Graphical abstract

The optical properties of antimony make it a promising candidate for photonic memory applications due to the large contrast between crystalline and amorphous solid states. This study resolves the entire switching cycle from crystal to glass and back. It elucidates the structural origin of the optical properties of the final and intermediate transient states, which are relevant for high bandwidth photonic applications.

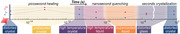

## 1 Introduction

The building blocks of integrated photonic circuits seek to control light on very different timescales. On the fast end, electro‐optic modulators of the light's amplitude or phase are reaching hundreds of gigahertz bandwidths,^[^ ^1^, ^2^ ^]^ enabling high‐speed data transmission and, more recently, also processing in non‐von Neumann computing concepts.^[^ ^3^, ^4^, ^5^ ^]^ On the other end, these novel computing concepts benefit from memory elements that can store information for long periods of time without continuous power consumption and without the overhead of converting between electrical and optical signals.^[^ ^3^, ^6^ ^]^ One such suitable optical memory has been identified in the form of small volumes of so‐called phase change materials (PCMs),^[^ ^7^, ^8^, ^9^, ^10^, ^11^, ^12^ ^]^ because of their large non‐volatile contrast in optical properties between ordered and disordered atomic structures and the ability to tune the relative volume fractions within nanoseconds.

PCMs are typically composed of germanium, antimony and tellurium. Their ordered, crystalline state is semiconducting or even semi‐metallic and exhibits a high refractive index and extinction coefficient in the visible and near infrared due to strong interband absorption. The disordered glass state is highly resistive with a markedly lower refractive index and extinction coefficient. The formation of the glass state requires the application of short optical or electrical pulses, which heat an initially crystalline PCM above its melting temperature, resulting in its transition to a metallic liquid phase. Subsequent rapid cooling through the supercooled liquid regime can avoid crystallization and instead leads to the formation of a semiconducting glass. A switching cycle is completed by recrystallization, which can typically be realized within nanoseconds by moderate heating.

Building on decades of research^[^ ^13^, ^14^, ^15^, ^16^, ^17^, ^18^ ^]^ and the development of optical as well as electronic memory based on PCMs (rewritable optical discs^[^ ^19^, ^20^ ^]^ and Intel's Optane chip, respectively), fundamental aspects of the large property contrasts between crystal, liquid and glass and the underlying nature of the chemical bond remain in scientific discourse.^[^ ^21^, ^22^, ^23^, ^24^, ^25^, ^26^ ^]^ Experimentally, a major challenge arises from the extremely high heating and cooling rates needed for amorphization, as transitions during the switching process are obscured by the limited time resolution of most experimental techniques. So far, ultrafast pump‐probe experiments were focused on short‐time (usually reversible) excitations and thus limited to a small excerpt of the multi‐scale response of PCMs.^[^ ^27^, ^28^, ^29^, ^30^, ^31^, ^32^, ^33^ ^]^ In particular, the property contrast associated with the melting transition and its evolution during the vast supercooled liquid regime remains yet unresolved.

Here, we resolve the complete switching cycle of a phase change material, spanning 15 orders of magnitude in time, with femtosecond resolution in stroboscopic optical pump‐probe measurements: from the large ultrafast photoinduced response on hundreds of femtoseconds timescales, lattice heating and melting within picoseconds, quenching through the supercooled liquid and glass formation over nanoseconds, all the way to recrystallization within minutes. We chose pure antimony, the stoichiometrically simplest PCM, as a model system.^[^ ^34^, ^35^, ^36^, ^37^ ^]^ The simplicity of the atomic structure and the fast structural dynamics of antimony allow us to compare directly the experimental results with ab‐initio molecular dynamics simulations of the quenching process. We find that the dissolution and formation of a structural distortion motif common to PCMs is contributing not only to the property contrast between distorted crystal and undistorted liquid, but that it is also the origin of the large optical response both in the ultrafast regime and during the transition from the metallic supercooled liquid to the semiconducting glass.

It becomes evident that at the operating bandwidths of photonic circuits, switching between two solid PCM states no longer constitutes a simple binary transition. Instead, it entails orders of magnitude in time of evolving properties through an intricate sequence of events. In the following, we will step by step resolve the response on the path to forming a glass, starting with the first picoseconds after excitation of crystalline antimony.

## 2 Structural Distortion and Electronic Structure

**Figure 1:**
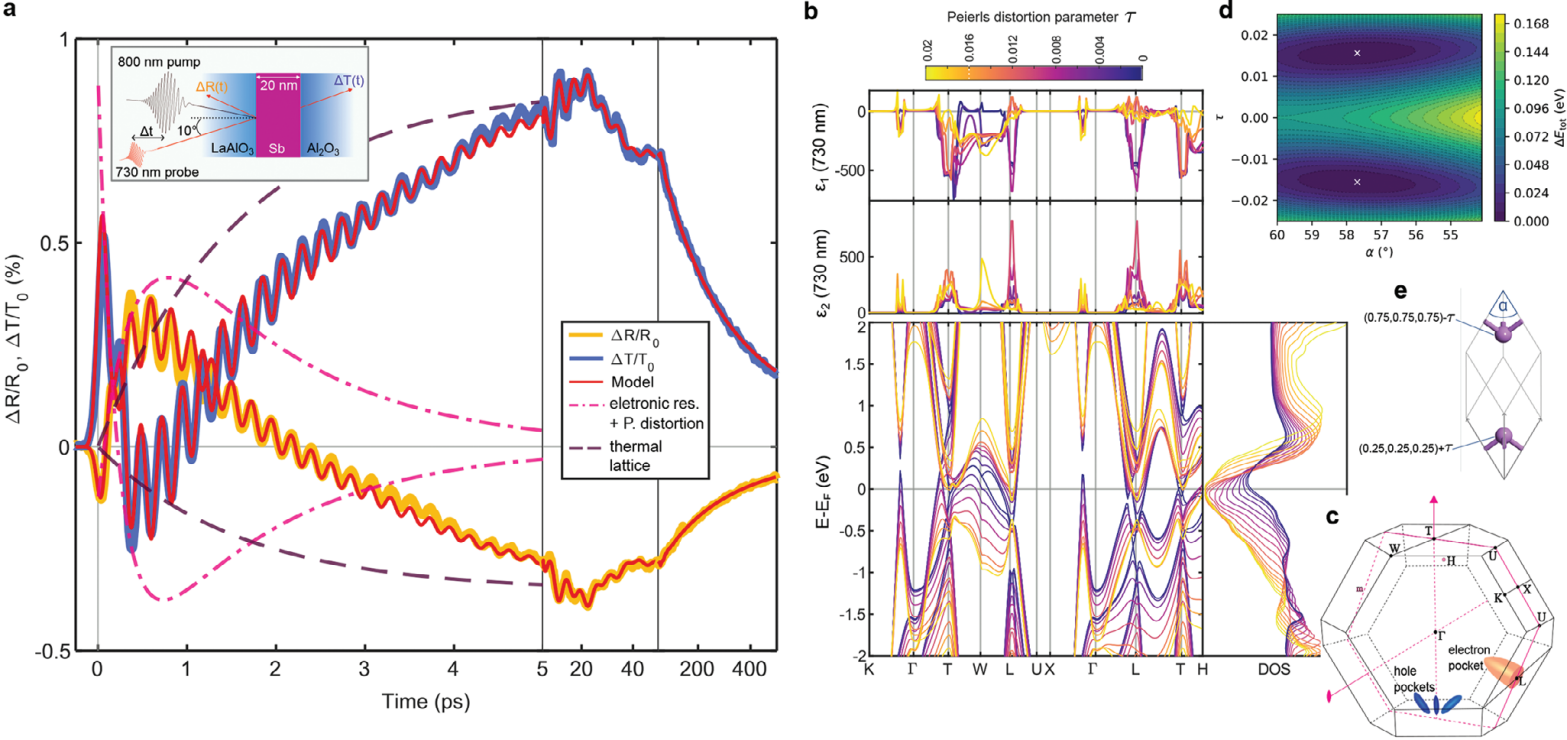
Photoinduced response and structure. a) Relative change in reflectance and transmittance of a 20 nm thick crystalline antimony film at 730 nm probing wavelength during the first 500 ps following excitation with a low fluence pump pulse. The red line corresponds to the fit of a phenomenological model. On short timescales, two components of the model, incoherent heating of the lattice and the combined electronic response and coherent structural shift, are also shown. The inset in the top left sketches the geometry of the experiment. b) (Bottom) Electronic band structure and density of states from DFT calculations for crystalline antimony structures with different Peierls distortion parameters τ. (Top) Corresponding ***k***‐resolved real and imaginary relative permittivity at 730 nm (1.7 eV). c) The first Brillouin zone with high symmetry points and the location of electron‐ and hole pockets (see e.g., ref. [38]). d) Potential energy landscape as a function of the two structural parameters, α and τ, shown in the rhombohedral unit cell of antimony in (e).

Antimony crystals are trigonal with the space group $R\bar{3}m$.^[^ ^39^, ^40^ ^]^ The two atoms in the rhombohedral primitive unit cell are displaced from the high‐symmetry positions by a so‐called Peierls distortion parameter τ, resulting in three short and three long nearest neighbor distances (**Figure** **1**e). Such a distortion motif is found in many materials with half‐filled *p*‐valence bands, i.e., group 15 elements, and PCMs in general.^[^ ^21^, ^41^, ^42^, ^43^, ^44^ ^]^ It is a consequence of a subtle energy balance between the different contributions to the potential energy landscape (see Supporting Figure S1, Supporting Information, for a breakdown into individual energy terms), which shifts the equilibrium atomic positions to τ = 0.016 for antimony (Figure 1d). The distortion's most significant consequence for the electronic properties is the opening of a bandgap around the Fermi level, which increases with the degree of distortion.

In simulations, the energy balance can be perturbed by increasing the temperature of the electron system (or equivalently the excited charge carrier density),^[^ ^45^, ^46^ ^]^ leading to the occupation of antibonding states in the conduction band which is accompanied by a shift in the equilibrium structure towards a lower distortion τ. As we are interested in the resulting changes to electronic states and optical properties, we calculate the electronic band structure and density of states for antimony with distortions ranging from slightly above that of the equilibrium structure to undistorted structures with τ = 0 (Figure 1b). With decreasing distortion valence band states shift upwards in energy, closing the bandgap, but also affecting states far from the Fermi level. Consequently, small changes in distortion have an influence on the joint density of states of the optical transitions, and hence the optical properties over a wide energy (wavelength) range from the visible to the IR. This is exemplified for the specific wavelength of 730 nm, in the calculated ***k***‐resolved real and imaginary permittivity contributions in the upper half of Figure 1b. At 730 nm and across the broad interband absorption peak of antimony, the main contributions to the permittivity are found in the Brillouin zone in the vicinity of the electron pockets at the *L* point and the hole pockets at the *H* point.

## 3 Sub‐Melting Threshold Response

In experiments, the temperature of the electron system can be increased by excitation with ultrashort laser pulses. Figure 1a shows the measured change in reflectance and transmittance at 730 nm of a crystalline antimony thin film during the first 500 ps, following excitation with a 60 fs pump pulse. The overall optical response can be described with a phenomenological model (red line in Figure 1a) that captures the time dependent distortion, the energy flow from the electron system to the atomic lattice on short timescales and heat dissipation to the surrounding on long timescales, together with damped sinusoidal oscillations at different frequencies. In the following, we will briefly discuss the main processes responsible for the optical response. At time = 0 the 800 nm pump pulse excites the electron system by promoting electrons from valence band states to the conduction band with an initial non‐thermal distribution, i.e., a charge carrier occupation function that cannot be described by a Fermi–Dirac distribution. The occupation of these states evolves rapidly by carrier–carrier interaction processes, and a quasi‐equilibrium situation is reached with a well defined electron temperature on a timescale of about 100 fs. Simultaneously, rapid ambipolar diffusion establishes a uniform spatial distribution of hot carriers in small material volumes, despite an initially inhomogenoues absorption profile. This sequence of events is rather general for semiconducting/semi‐metallic materials.^[^ ^47^, ^48^, ^49^, ^50^, ^51^ ^]^

In antimony and other half‐filled *p*‐band materials, there is an additional process on these sub‐picosecond timescales. As a response to the impulsive increase in temperature of the electron system (i.e., the excitation of electrons from bonding to antibonding states), atoms collectively move to their new equilibrium positions with a lower distortion τ, and oscillate coherently around the new minimum with a zone center (A_1g_) optical phonon mode.^[^ ^52^ ^]^ This mechanism is therefore called displacive excitation of coherent phonons. The damped sinusoidal 4.4 THz oscillations (with a small chirp) around a new quasi‐equilibrium distortion modulate the optical response (observable in the first 5 ps in Figure 1a; Figure S2, Supporting Information). This intricate coupling between the electron system and the A_1g_ mode is widely studied in ultrafast pump‐probe experiments.^[^ ^53^, ^54^, ^55^, ^56^, ^57^ ^]^

Here we will concentrate on the optical contrast that results already from a small shift in the equilibrium position. After thermalization of the electron system during which the change in reflectance (transmittance) changes its sign, the measured signal assumes a maximum (minimum) at around 500 fs that is largely governed by the shift in distortion. This aspect will be further discussed in the next section, where we compare measured and calculated optical properties. Electron‐phonon coupling subsequently determines the time evolution of the response. Cooling of the electron temperature returns the distortion parameter to its equilibrium value, heating the atomic lattice in the process, which is shown as a dashed line in Figure 1a. The magnitude of the distortion τ is independent of lattice temperature in antimony up to the melting point.^[^ ^39^, ^40^ ^]^ The time constant for electron‐phonon coupling of about 3 ps extracted from the optical response agrees with more direct measurements of a rising lattice temperature in time‐resolved diffraction experiments on antimony.^[^ ^58^ ^]^ As the antimony film heats up, it expands, exciting breathing‐mode‐like strain oscillations, and launching strain waves into the oxide capping (and substrate). Over the following 50 ps, the optical response is governed by these strain oscillations with frequencies determined by the film thicknesses and the respective sound velocities.

**Figure 2:**
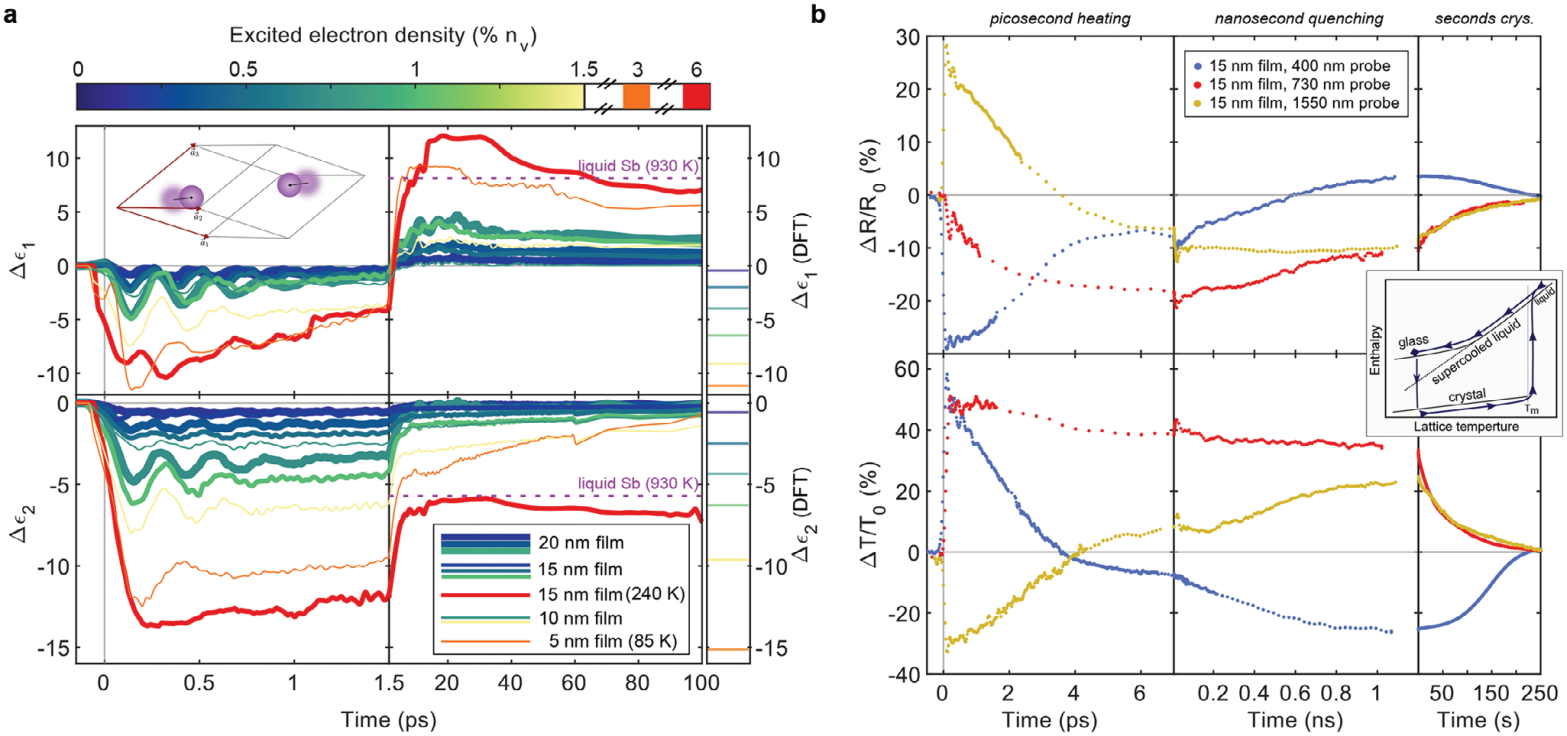
Permittivity contrast and cycling. a) Change in real and imaginary relative permittivity during the first 100 ps after photoexcitation of crystalline Sb films. Due to the initially non‐uniform absorption profile of the pump pulse, there are two ways to tune the excited electron density (temperature of the electron system) in experiments: directly by the pump pulse fluence or by the antimony film thickness. For short timescales and electron densities up to 1.5 %, the response is well described by a shift in distortion τ as illustrated by the calculated change in permittivity on the right. The response with 6 % excited electron density transitions to the liquid state. The pink dashed line for liquid antimony at 930 K is estimated from Hodgson et al.^[^ ^59^ ^]^ b) Stroboscopic (low duty cycle) measurement of the entire switching cycle at three different probing wavelengths. For each data point on picosecond and nanosecond timescales, the 15 nm film goes through the entire switching cycle, where each subsequent individual high fluence pump pulse is only applied after complete recrystallization. The inset on the right illustrates the path to glass formation and its subsequent recrystallization.

**Figure 3:**
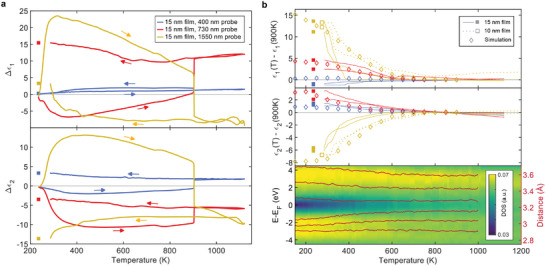
Permittivity contrast and supercooled liquid. a) Change in real and imaginary relative permittivity with respect to initially crystalline films at three different probing wavelengths as a function of temperature. During cooling the system does not quite reach the base temperature of 240 K. The next data point, acquired one millisecond later, is shown as a solid square. b) (Top) Comparing the real and imaginary permittivity from experiment and ab‐initio molecular dynamics simulations during quenching through the supercooled regime of antimony. The measurement is repeated multiple times on different spots on the 15 nm film and on a different sample with 10 nm film thickness to check the reproducibility of the experimental results. The simulated data is acquired over five molecular dynamics snapshots and averaged. Both simulated and experimental data are smoothed over a 100 K sliding window. (Bottom) Mean of the distributions of the six nearest neighbor distances together with the electronic density of states as a function of temperature.

For excitations with pump pulse energies below the melting threshold, the antimony film returns to equilibrium within a nanosecond by heat dissipation to capping and substrate. This process is limited by the respective thermal interface conductances, as is further discussed in the context of two‐temperature modeling in the Experimental Section and in Figure S4 (Supporting Information). As a consequence, no temperature gradients form within the antimony film during cooling. For the largest part of the response, after rapid ambipolar diffusion establishes a homogeneous distribution of hot carriers within 100 fs, it is reasonable to assume a thin film with uniform optical properties. We can therefore use a transfer‐matrix method to extract transient permittivities from the measured change in reflectance and transmittance. The approach is further discussed in the Experimental Section and the results are presented in **Figures** **2** and **3** below.

## 4 Structural Distortion and Permittivity Contrast

In the following we increase the excited electron density in the experiment, ultimately going beyond the melting threshold. Figure 2a shows the time evolution of the change in the relative permittivity at 730 nm for the different degrees of excitation. On short timescales, both real and imaginary part of the permittivity decrease. For higher excited electron densities, the magnitude of this permittivity offset increases and the oscillations of the A_1g_ mode around this new quasi‐equilibrium value become more pronounced (while softening and decreasing in lifetime). In Figure 2a we also compare the measured permittivity change on these sub‐picosecond timescales for different excited electron densities with the calculated permittivity change based on electronic structure calculations for different distorted structures (shown in Figure 1b. This requires mapping the experimentally realized excited electron densities to the corresponding structural distortions (the mapping function is shown in Figure S3, Supporting Information).^[^ ^45^, ^46^ ^]^ For excited electron densities up to 1.5%, the calculated permittivity contrasts agree very well with the measured values in the sub‐picosecond regime. For higher excitation densities, the calculation overestimates the response, which can be explained by the fact that the experimentally observed state after about 500 fs is not purely characterized by a decrease in distortion. Electron‐phonon coupling, with its 3 ps time constant, already affects the measured optical response on these timescales, since it brings the distortion back into equilibrium while heating the lattice, driving the change in the imaginary part of the permittivity to lower values and eventually reversing the sign in the real part. With this in mind, a simple shift in distortion can capture the main aspect of the photoinduced response during the first picosecond after excitation. Thus, we understand the contrast in optical properties on these ultrafast timescales to be the consequence of a change in the electronic structure that originates from a coherent shift towards an undistorted atomic structure. The universal nature of this structural response to photoexcitation in crystalline PCMs on ultrafast timescales is supported by time‐resolved diffraction experiments on Ge_2_Sb_2_Te_5_aand time‐dependent density functional theory simulations of GeTe.^[^ ^33^, ^60^ ^]^ This explanation is in contrast to earlier interpretations, which identified a depletion of bonding states and thus a change in the occupation of electronic states,^[^ ^29^, ^31^ ^]^ rather than a change in electronic structure, as the origin of the large ultrafast response of PCMs.

At the 730 nm probing wavelength the main effect of the subsequent increase in lattice temperature on a few picoseconds timescale can be interpreted by a reduction in the overlap of molecular orbitals due to an expanding lattice and thermal disorder. Consequently the interband transition rate decreases as can be seen in the (small) negative change in the imaginary part.

## 5 Resolving the Entire Switching Cycle

All antimony films in Figure 2a with excited electron densities up to 3 % of valence electrons remain below the melting threshold and therefore return to the equilibrium crystalline state within nanoseconds. The 15 nm film with a 6 % excited electron density, however, melts and eventually quenches to the glass state. On picosecond timescales, this results in a departure from the sub‐melting threshold response. This is clearly seen in the imaginary part ϵ_2_ that initially follows a similar trajectory to the sub‐melting threshold excitations, but departs during heating of the lattice and remains just below the imaginary permittivity of liquid antimony (estimated from ref. [59]). Figure 2b shows the corresponding change in reflectance and transmittance at 730 nm together with measurements at 1550 nm and 400 nm probing wavelengths, i.e., close to the maximum of interband absorption in antimony and at its high energy tail. The measured and calculated (static) permittivities of crystalline antimony are shown in the Figures S5 and S6 (Supporting Information) respectively.

In the initial picoseconds following excitation with a high‐fluence pump pulse, the optical response is governed by the same processes as for low‐fluence excitations below the melting threshold. Strongly damped coherent A_1g_ mode oscillations are observed on top of a large (relative) change in transmittance and reflectance for all three probe wavelengths. This clearly demonstrates that the film has not undergone non‐thermal melting, but instead remains crystalline on these timescales. The lattice temperature rises through electron‐phonon coupling with a 3 ps time constant, which shifts the distortion parameter τ back toward its equilibrium value, again accompanied by large changes in the optical response. The crystalline antimony film eventually melts, though the transition to the liquid state spans several picoseconds and is therefore less sharp than in the temperature domain plot below. During the next 100 ps, the measured signals initially remain approximately constant as the system cools. The signals start to evolve again at about 200 ps until remaining constant for several milliseconds after 1 ns.

The fact that the crystalline antimony film melts, quenches through the supercooled liquid regime and forms a glass within a nanosecond is evidenced by the fact that the optical properties do not recover the equilibrium crystalline values on the nanosecond timescale of thermal dissipation to substrate and capping, but only through (slow) recrystallization (at the experimental base temperature of 240 K) after minutes. The glass state could even be stabilized at room temperature by confining antimony into thinner film thicknesses,^[^ ^34^, ^37^, ^61^, ^62^ ^]^ though this would be impractical for our stroboscopic measurement approach that repeatedly probes the same sample volume.

We calculate the transient permittivities from the measured signals as in the sub‐melting threshold experiment. Additionally, we compare quenching through the supercooled liquid regime of antimony with ab‐initio molecular dynamics simulations of the same process to resolve the atomic structure throughout. To translate the time dependence of the permittivity evolution to a temperature dependence, we model the heating, melting and quenching process using a two‐temperature model based on Shin et al.^[^ ^51^ ^]^ The details of the model are discussed in the Experimental Section and in Figure S4 (Supporting Information).

**Figure** **3**a shows the evolution of the permittivity during the switching cycle now as a function of temperature, yet omitting the isothermal recrystallization step on a timescale of seconds to minutes. The largest optical contrast for all wavelengths is observed right after excitation, i.e. in the undistorted crystalline state, before cooling of the electron system increases the distortion again. The melting transition at 904 K leads to a marked step‐like change in permittivity in the opposite direction of the ultrafast response. However, the liquid at the melting temperature has a coordination number of 6 with little (if any) distortion,^[^ ^63^, ^64^ ^]^ and consequently an electronic density of states that is similar to the undistorted crystalline state at high electron temperatures, i.e., with a large density of states at the Fermi level.^[^ ^65^, ^66^ ^]^ Therefore, a change in the joint density of states alone cannot explain the observed optical contrast upon melting and a second puzzle piece is missing.

As proposed by Huang et al.,^[^ ^22^ ^]^ disordering the atomic lattice of a PCM disrupts the aligned rows of *p*‐orbitals of the crystalline state. This greatly reduces thereby enhanced matrix elements of the optical transitions, which can explain the large optical contrast between crystal and glass, despite very similar electronic densities of states. For the melting transition between non‐equilibrium crystal and (high‐temperature) disordered liquid, one must expect that both a change in joint density of states and matrix element contribute to the optical contrast. The latter at least seems to dominate the response at 1550 nm, close to the maximum of the interband absorption peak(s). Upon melting, the imaginary part of the relative permittivity drops by almost fifteen, whereas the formation of an undistorted, but ordered crystalline state on ultrashort timescales is driving the response in the opposite direction. A free electron contribution through a Drude term does not seem to play a major role in either case, since it would lead to an increase in the imaginary part of the permittivity while simultaneously decreasing its real part.

Following the melting transition, there remains some excess energy in the electron system, so the lattice temperature continues to increase to about 1200 K. There is almost no change in optical properties during the subsequent quenching of the liquid, even down to 300 K below the melting temperature (most clearly visible at 1550 nm probing wavelength). The final contrast that is ultimately observed between crystal and glass only starts to evolve at an onset temperature of around 600 K. To understand this, we simulate the quenching process through the supercooled liquid regime in antimony using ab‐initio molecular dynamics. The top of Figure 3b compares the evolution of the permittivity, relative to its respective value at the melting temperature in experiment and simulation. Both onset temperature and magnitude of shift in permittivity agree fairly well across the three probing wavelengths. Furthermore, the continuous evolution in permittivity with decreasing temperature seems to stop, or at least slow down, at around the same temperature of just over 300 K, which might be explained by the drastic, highly non‐linear slowing down of structural relaxation processes close to a glass transition. The lower part of Figure 3b explains the origin of this final change in optical properties on the way to forming a glass. Starting again at around 600 K, the mean of the distribution of the six nearest neighbor distances evolves towards the coordination of the equilibrium crystal, with its three short and three long nearest neighbor distances. This can be interpreted as an increase in the Peierls‐like, i.e., Jahn–Teller,^[^ ^21^ ^]^ distortion motif that also characterizes the crystal (see Figure S7, Supporting Information, for more details). Again, the universal nature of this increase in a Peierls‐like distortion motif in the supercooled liquid of PCMs is supported by time‐resolved diffraction experiments and simulations of a different PCM.^[^ ^67^ ^]^ Simultaneously, the electronic density of states evolves towards that of the equilibrium crystal, as the density of states at the Fermi level decreases and a pseudo‐gap opens, resulting in a blue shift of optical transitions as evidenced by a decrease of the imaginary permittivity in the near IR and and increase in the visible (also see Figure S6c,d, Supporting Information). In this sense, the final change in optical properties about one nanosecond after excitation mirrors the first feature on ultrafast timescales: A decrease in distortion closes a pseudo‐(band)gap and an increase in distortion opens it again, thereby strongly affecting the optical properties across a broad wavelength range.

**Figure 4:**
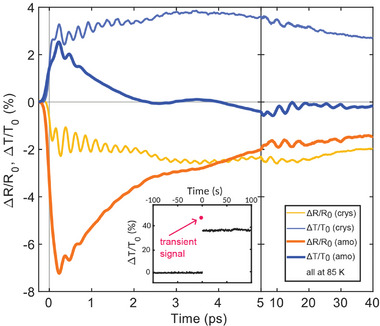
Response of the amorphous film. Relative change in reflectance and transmittance at 730 nm during the first 40 ps of a antimony glass film and a crystalline film, both with thickness of 10 nm and at a base temperature of 85 K. The two states are excited with the same electron density, accounting for the lower absorption of the glass film. The inset shows the amorphization process, where a single, high fluence pump pulse is applied at time = 0 (seconds) while continuously (with 1 kHz) monitoring the transmittance with the probing pulse. This measurement scheme affords one transient data point with femtosecond temporal resolution for each arrival of a pump pulse, which is the principle behind the stroboscopic measurements of the switching cycle at elevated temperature in Figure 3. The transmittance increases by more than 35%. At this low base temperature, the film does not recrystallize over days.

We note that a temperature dependent modification in the short‐range order accompanied by an opening of a pseudo‐gap is not unique to PCMs, but observed in many systems with metallic high temperature liquids and semiconducting glasses (e.g., germanium, silicon or tellurium).^[^ ^68^ ^]^ However, the short‐range order in antimony glass, and likely in glassy states of other materials with half‐filled *p*‐valence orbitals, is characterized by its particular energy balance between the different contributions to the potential energy landscape that drive the system in opposite directions. Perturbing this energy balance through impulsive excitation of the electron system results in an ultrafast response that is astonishingly similar to what is observed in the crystalline film **Figure** **4**). The atomic structure collectively evolves towards a lower distortion, which is accompanied by a large change in optical properties. The impulsive shift in the equilibrium atomic coordinates excites a vibrational mode with the same phase and frequency as the A_1g_ phonon mode in the crystalline film, but with a much shorter lifetime.

## 6 Conclusion

We resolve the entire photoinduced switching cycle of the phase change material antimony. On ultrafast timescales, the excitation of charge carriers to antibonding states results in a shift in the quasi‐equilibrium atomic structure towards a smaller Peierls distortion. Density functional theory calculations demonstrate the (energetically) wide‐ranging consequences for the electronic structure, reaching far below and far above the Fermi level. As a result, a small shift in distortion strongly influences the optical properties across the entire interband absorption region, from the visible to IR. As energy is transferred from the hot electron–hole system to the lattice, it melts after few picoseconds, initially transitioning to an undistorted, metallic liquid. The fast structural dynamics deep into the supercooled liquid of antimony, combined with the high experimental quenching rates, result in an ab‐initio simulated response that seems to fall out of equilibrium at a similar temperature as the experimentally measured. Before, over hundreds of Kelvin, there is very little change in the optical properties and in the atomic and electronic structure accordingly. Starting at an onset temperature of around 600 K, it is again the formation of Peierls‐like distortions that contributes decisively to the optical contrast that is observed on long timescales between crystal and glass.

It is evident that the switching of antimony involves multiple intricate processes through intermediate transient states. These states are characterized by distinct optical properties, with larger optical contrasts than the one usually exploited between the non‐volatile crystalline and amorphous states of PCMs. For the application of PCMs as optical memory in high‐bandwidth photonic circuits, setting a certain memory level is therefore inevitably accompanied by these orders of magnitude in time spanning dynamics. One can imagine new functionality that is actually based on this volatile change in permittivity. T. Y. Teo et al. for instance implemented an all‐optical on‐chip nonlinear activation function by optically exciting a patch of PCM on a microring resonator with fluences below the melting threshold.^[^ ^69^ ^]^

Based on the understanding and quantification of the ultrafast response of PCMs provided here, they can be exploited in high‐bandwidth photonic applications beyond non‐volatile memory. New types of materials beyond classical PCMs can be identified that exhibit a similar distortion motif, but where their poor glass‐forming capability has so far precluded application.

## 7 Experimental Section

### Sample Fabrication

Antimony thin films of four different thicknesses (5 nm, 10 nm, 15 nm and 20 nm) were deposited on c‐plane sapphire substrates and capped with 50 nm LaAlO_3_ using pulsed laser deposition. The fabrication process is described in greater detail in Yimam et al.^[^ ^37^ ^]^ The crystalline nature of the films before the following experiments was ensured by annealing the samples at 450 K under vacuum for 5 h.

The main consequence of reducing the film thickness in this range for the material properties of antimony is a non‐linear reduction in the crystallization velocity. While a 200 µm diameter amorphous spot crystallizes within 3 min in the 15 nm film at a base temperature of 240 K, it takes 30 min for the 10 nm film at the same temperature and the 5 nm film was even stable at room temperature for hours.

For optical excitation with sub‐melting threshold fluences even prolonged exposure over days (at 500 Hz repetition rate) does neither alter the static optical properties nor the amplitude and time constants of the photoinduced response, which would also be sensitive to changes in the thermal dissipation time constant over time. At the same time, the photoinduced response across all investigated film thickness is identical beyond a trivial scaling factor, i.e., we do not observe a possible larger influence of an interface region in the thinner films.

### Optical Measurements

The (static) optical properties of the antimony films were determined by ellipsometer measurements (Woolam, M‐2000) over a wavelength range from 370 nm to 1690 nm. The measurement results were fitted using the transfer‐matrix method, where all model parameters were fixed except for the complex refractive index of antimony, that is parameterized using Kramers–Kronig consistent splines as implemented in the manufacturer's “CompleteEASE” software. The measured optical properties of antimony agree with literature data (see Figure S5, Supporting Information, for a comparison).

The time‐resolved measurements were performed in an ultrafast optical pump‐probe setup. The sample was placed in a liquid nitrogen cooled cryostat (Advanced Research Systems) for temperature control. Laser pulses were generated in an amplified Ti:Sapphire system (Coherent, Astrella) that emits 800 nm, 57 fs pulses with a repetition rate of 1000 Hz. A 50:50 beam splitter separates the output in pump and probe pulses. The pump pulses were chopped to 500 Hz, attenuated using a λ/2‐plate and Glan calcite polarizer, delayed by a mechanical delay‐line, and focused onto the sample at near normal incidence to a 200 µm diameter spot size determined by the the knife‐edge method. The wavelength of the probe pulse was set with an optical parametric amplifier (Light conversions, TOPAS‐Prime). It was attenuated to below 100 nJ per pulse using optical densities. A fraction of the probe pulses was split off and recorded at a reference photodiode to account for fluctuation in the laser output and improve the signal‐to‐noise ratio. The probe pulses were then focused onto the sample to a 20 µm diameter spot size and the reflected or transmitted pulses were recorded at a second photodiode, amplified and low‐pass filtered (FEMTO, DLPCA‐200) and digitized using a data acquisition card.

For the stroboscopic sub‐melting threshold excitation experiments, the sample was pumped at a 500 Hz repetition rate and probed at 1000 Hz in a detection scheme similar to the one described by Werley et al.^[^ ^70^ ^]^ At each time‐delay between pump and probe set by the delay line, the signal was averaged for 2 s. Due to the small pumped volume and good thermal conductivity of the substrate, the system returns to equilibrium within few nanoseconds. Cumulative heating effects can therefore be excluded.

For the stroboscopic switching experiments the sample was pumped at a much lower rate, i.e., every 2 min, to accommodate the return to equilibrium by the comparatively slow recrystallization. The base temperature is lowered to 240 K (270 K) in these experiments to be able to exclude recrystallization effects on nanosecond timescales. An individual pump pulse is selected from the 500 Hz pulse train with a fast mechanical shutter (based on the actuator arm of a hard disk drive). In its closed state, the shutter blocks the pump pulse. A pulse generator synchronized with the laser output applies a voltage pulse to the coil of the actuator that opens and closes sufficiently fast to only let a single pump pulse pass, while the sample is continuously (with 1000 Hz) probed. This scheme allows for the measurement of a single transient data point with femtosecond time resolution for each switching cycle. The data points in Figure 2b are hence acquired without any averaging. The measurement was repeated multiple times, scanning the time‐delays forward as well as backward, to exclude undetected cumulative sample degradation altering the photoinduced response over the few hundred pump pulses.

### Phenomenological Model

The photoinduced transient change in transmittance and reflectance (and as such the change in complex permittivity) can be described by a phenomenological model (see red line in Figure 1a) that consists of the following components *S*_*i*_. On short timescales, immediately after excitation at time *t* = 0, the signal is shaped by the thermalization of the hot electron–hole plasma and the formation of a quasi‐equilibrium atomic structure with a lower distortion,

(1) $$\begin{array}{c} S_{1}=\left[A_{\text{non-thermal}}+A_{\text{eh+distortion}}\left(1-\exp\left(-t/\tau_{\text{th}}\right)\right)\right]\cdot \exp\left(-t/\tau_{\text{ep}}\right) \end{array}$$

Here, *A*_non‐thermal_ is the amplitude of the response before carrier‐carrier scattering can establish a thermal distribution with time constant τ_th_ and amplitude *A*_eh + distortion_, while simultaneously energy is transferred to the lattice with the electron–phonon time constant τ_ep_. The impulsive shift in the equilibrium position of atoms leads to the displacive excitation of chirped coherent A_1g_ vibrational modes with amplitude *A*_A1g_ and damping time constant τ_A1g_,

(2) $$\begin{array}{c} S_{2}=A_{\text{A1g}}\cos\left(\omega_{\text{A1g}}t+\phi_{\text{A1g}}\right)\cdot \exp\left(-t/\tau_{\text{A1g}}\right) \end{array}$$

where ϕ_A1g_ is the phase offset of the vibration (close to zero for the displacive excitation mechanism) and ω_A1g_ is the instantaneous frequency given by

(3) $$\begin{array}{c} \omega_{\text{A1g}}=2\pi \cdot 4.4\mathrm{THz}-A_{\text{chirp}}\exp\left(-t/\tau_{\text{ep}}\right) \end{array}$$

The temperature increase of the lattice on short timescales and the subsequent heat dissipation to capping and substrate is described by

(4) $$\begin{array}{cc} S_{3}=\; & A_{\text{lattice}}\left(1-\exp\left(-t/\tau_{\text{ep}}\right)\right)\\ & \cdot \left[A_{\text{substrate}}\exp\left(-t/\tau_{\text{substrate}}\right)+\left(1-A_{\text{substrate}}\right)\exp\left(-t/\tau_{\text{capping}}\right)\right] \end{array}$$

Here *A*_lattice_ is the amplitude of the lattice response, *A*_substrate_ corresponds to the heat fraction that is dissipated to the substrate with time constant τ_substrate_ and τ_capping_ is the corresponding time constant for the dissipation to the capping. Finally, the rapid heating and expansion of the lattice launches strain waves that modulate the signal on timescales of tens to hundreds of picoseconds with three characteristic frequencies,

(5) $$\begin{array}{c} S_{4}=\sum_{n=1}^{3}A_{n}\cdot \cos\left(\omega_{n}t+\phi_{n}\right)\cdot \left(1-\exp\left(-t/\tau_{\text{ep}}\right)\right)\cdot \exp\left(-t/\tau_{n}\right), \end{array}$$

where *A*_*n*_, ω_*n*_ ϕ_*n*_ and τ_*n*_ are the amplitude, frequency, phase and damping time constant of the respective strain oscillation.

The final model is the sum of the individual components *S*_*i*_, multiplied with a step function and convoluted with the instrument response function (a Gaussian with τ = 85fs FWHM). The change in reflectance and change in transmittance response were fitted simultaneously using different amplitudes *A*, but identical time constants τ and phases ϕ, with a gradient descent algorithm as implemented in Matlab's “fmincon” function. This minimal component phenomenological model can describe the sub‐melting threshold response of the four different samples across a broad fluence range and at a base temperature from 85 K to 450 K.

### Time Dependent Transfer‐Matrix Method

Transient permittivities were extracted from the measured relative change in reflectance and transmittance using a time dependent transfer‐matrix method. Briefly, in the transfer‐matrix method the light wave is propagated through the experimental layer stack by dividing the space into a sequence of uniform regions, and by deriving a scattering matrix based on the Fresnel equations that relates the values of the fields at each interface.^[^ ^71^ ^]^

The assumption was made that only the optical properties of the antimony film change following photoexcitation and that this change is homogeneous throughout the film. Based on the known film thicknesses of the layer stack and the measured static optical properties, the measured relative change is described in reflectance and transmittance using the transfer‐matrix method and a time dependent change in real and imaginary relative permittivity as the fit parameters. In these ultrafast experiments, there is an additional complication because of the propagation delay introduced by the comparatively thick (300) substrate, which is larger than both the pulse length and the typical timescale of the photoinduced processes. A probing pulse that arrives at the sample at some time delay with respect to the pump pulse is sensitive to the time dependent optical properties at this instance. A fraction of the pulse gets transmitted through the antimony film and continues to propagate through the substrate until it eventually (after 1.7 ps) reaches the substrate—vacuum interface, where a small fraction gets reflected back towards the top of the layer stack. When the probe pulse arrives back at the antimony film, the transient optical properties have advanced in their time evolution by in total 3.4 ps. A fraction of the pulse again gets transmitted and adds to the measured intensity at the photodiode that records the reflected signal. A fraction gets reflected and adds to the measured intensity at the photodiode that records the transmitted signal.

This effect is most clearly seen at negative times (the probe pulse first arrives at the sample before the pump pulse) in the 5 nm and 10 nm samples, where a larger fraction of the probe pulse is initially transmitted through the antimony layer. As a consequence of the second delayed arrival of a fraction of the probe pulse, the change in reflectance signal has an onset at −3.4 ps that is simply a scaled copy of the change in transmittance signal that has its onset at *t* = 0. Likewise, the change in transmittance signal shows a scaled copy of the change in reflectance signal with an onset at −3.4ps.

Consequently, the measured signals are the result of a time dependent permittivity with contributions from (at least) two instances in time (*t*_1_ and *t*_2_ = *t*_1_ + 3.4ps). To account for this, the probing pulse was propagated first by the transfer‐matrix method through a layer stack, where the optical properties are described by permittivity at *t*_1_ as a fit parameter, and assuming an infinitely thick substrate layer. The reflected amplitude at the substrate‐vacuum interface was then calculated in a separate step and the reflected pulse was propagated backwards through the layer stack, where the optical properties of the antimony film are now described by the permittivity at *t*_2_. This fitting procedure in the case of the 5 nm film leads to a shift in the extracted transient change in permittivity of at most about 8 % compared to a simpler procedure with independent permittivities in time. In principle even multiple reflections at the substrate‐vacuum interface could play a role, though their contribution to the measured signals is negligible.

### Two‐Temperature Modeling

To calculate the time‐dependent lattice temperature during the switching process, a two‐temperature model was employed based on the one developed by Shin et al.^[^ ^51^ ^]^ for bismuth (and semi‐metallic or semiconducting materials in general). The model describes the photoinduced response through three coupled diffusion equations that describe the temporal and spatial evolution of the excited electron density *n*_*e*_, electron temperature *T*_*e*_ and lattice temperature *T*_*i*_,

(6) $$\begin{array}{cc} \frac{\partial n_{e}}{\partial t} & =D_{e}\frac{\partial^{2}n_{e}}{\partial z^{2}}-\frac{n_{e}-n_{0}}{\tau_{r}}+{\dot{n}}_{s},\\ n_{e}C_{e}\frac{\partial T_{e}}{\partial t} & =C_{e}D_{e}\frac{\partial n_{e}}{\partial z}\frac{\partial T_{e}}{\partial z}-g_{0}n_{e}\left(T_{e}-T_{i}\right)+\left(\hbar \omega -E_{g}\right){\dot{n}}_{s},\\ n_{i}C_{i}\frac{\partial T_{i}}{\partial t} & =\kappa_{i}\frac{\partial^{2}T_{i}}{\partial z^{2}}+g_{0}n_{e}\left(T_{e}-T_{i}\right)+\frac{\left(n_{e}-n_{0}\right)\left(C_{e}T_{e}+E_{g}\right)}{\tau_{r}} \end{array}$$

Here, *D*_*e*_ = 40 cm^2^s^−1^ is the ambipolar diffusivity of charge carriers (for bismuth^[^ ^50^ ^]^), *n*_0_ = 6 × 10^20^ cm^−3^ is the equilibrium electron density in the conduction band^[^ ^72^ ^]^, τ_*r*_ = 10 ps is the electron‐hole recombination time constant (for bismuth^[^ ^50^ ^]^), *C*_*e*_ is the heat capacity of charge carriers (parameterized in [58]), *g*_0_ is the electron‐phonon coupling strength (parameterized in [58]), ℏω the pump photon energy, *E*_*g*_ = 0.2 eV is the band gap, *n*_*i*_ = 6.7g cm^−3^ is the atomic density, *C*_*i*_ = 0.21 J g^−1^ K^−1^ is the lattice heat capacity, κ_*i*_ = 18 W m^−1^ K^−1^ is the lattice thermal conductivity and ${\dot{n}}_{s}$ is the time dependent excited electron density source term.

The following assumptions and simplifications were made. The geometry was treated as 1D due to the large diameter of the pump pulse compared to the layer thicknesses and the smaller (by a factor ten) probe pulse diameter. Instead of describing the excitation with a time‐dependent source term ${\dot{n}}_{s}$, the excited electron density profile was calculated due to absorption of the ultrashort pump pulse using the transfer‐matrix method, which then serves as the starting condition of the model. The hot electrons were confined to the antimony layer, as it is encapsulated by large bandgap materials on either side with no overlap in their electronic densities of states around the Fermi level. The thermal interface conductances determine the lattice heat flow across the antimony‐capping and the antimony‐substrate interface and as such the cooling on long timescales. Its values are chosen so that the two‐temperature model can describe the cooling in the sub‐melting threshold experiments, in a fluence range where the measured signal depends linearly on lattice temperature. Lattice heating from charge carrier recombination is negligible in the model due to its longer time constant compared to the electron‐phonon coupling term and the semi‐metallic nature of antimony, as was also observed for bismuth and indirectly for antimony in refs. [50, 58] The separation of charge carriers by a bandgap also becomes questionable upon transitioning to the undistorted crystalline or to the liquid metallic state. The melting transition was modeled in the following way. Upon reaching the melting (lattice) temperature in some discretized volume element, further energy flow into this volume element no longer leads to an increase in lattice temperature. Instead the energy is integrated until the melting enthalpy (1092 × 10^−21^J nm^−3^) is reached and the volume element transitions to the liquid state.

The differential equations were solved using a forward time‐centered space scheme. An exemplary result is shown in Figure S4 (Supporting Information).

### Potential Energy Landscape for Crystalline Antimony in Dependence of Peierls Distortion

The potential energy of crystalline antimony was calculated in dependence of cell angle and Peierls distortion parameter using the Quantum Espresso code. The projector augmented wave method^[^ ^73^ ^]^ was used with fully relativistic Kresse–Joubert pseudopotentials,^[^ ^74^ ^]^ including spin‐orbit coupling effects. The exchange‐correlation functional was described in the approximation by Perdew, Burke, and Ernzerhof (PBE). The energy cutoffs for the wave function and the charge density were set to 30 Ha and 151 Ha, respectively. The wave function was sampled on a 15 × 15 × 15 grid of ***k***‐points. The occupation was smeared using Methfessel–Paxton smearing with a width of 0.01 Ha. Van der Waals effects were included using a Grimme D3 correction.^[^ ^75^ ^]^

### Ab‐initio Molecular Dynamics Simulations

The 2nd generation Car–Parrinello method was used as implemented in the Quickstep code^[^ ^76^ ^]^ of CP2k^[^ ^77^ ^]^ for all the ab‐initio molecular dynamics simulations. The scheme combines the efficiency of Car–Parrinello simulations with the large time steps used in Born–Oppenheimer molecular dynamics. A Langevin thermostat was employed to control temperature. The time step was set to 2 fs. The number of antimony atoms in the cubic simulation cell was kept constant at 728. Finite size effects can significantly influence dynamics in the system and have been discussed in a previous work.^[^ ^34^ ^]^ We quenched a model of antimony at the liquid density of 6.49 gcm^−3^ from the melt with a quenching rate of 9.5 K/ps.

### Calculation of Optical Spectra

The Kubo–Greenwood formula was used

(7) $$\begin{array}{ccc} \chi_{ij}\left(\omega \right) & = & \frac{e^{2}}{\hbar m_{e}^{2}V}\sum_{nmk}\frac{f_{mk}-f_{nk}}{\omega_{nm}^{2}\left(k\right)\left[\omega_{nm}\left(k\right)-\omega -i\Gamma /\hbar \right]}\\ & & \times p_{nm}^{i}\left(k\right)p_{mn}^{j}\left(k\right) \end{array}$$

as implemented in the QuantumATK software^[^ ^78^ ^]^ to calculate optical spectra from the ground‐state electronic wave function. Here, *e* and *m*_*e*_ are electron charge and mass, respectively, *V* is the unit cell volume, *f*_*m* **k**_ is the occupation of state *m* at ***k***‐point ***k***, ℏω_*nm*_(**k**) = *E*_*n*_(**k**) − *E*_*m*_(**k**) is the energy difference between two states, Γ is the energy broadening, and $p_{nm}^{i}=\langle nk\left|p^{i}\right|mk\rangle$ are components of the momentum operator.

To calculate optical properties for single snapshots from the molecular dynamics trajectory, we employed the PseudoDojo pseudo‐potential^[^ ^79^ ^]^ with a Linear Combination of Atomic Orbitals (LCAO) basis set of the “High” quality. The PBE exchange‐correlation functional was again used. 3 × 3 × 3 ***k***‐points per unit cell are used to accurately sample the electronic structure. The density mesh cutoff was set to 80 Ha. We calculated optical spectra using the Kubo–Greenwood formula for the susceptibility tensor^[^ ^80^ ^]^ with a single ***k***‐point in the reciprocal cell. The diagonal components of the permittivity tensor are averaged for comparison with experimental results of the liquid/glass and polycrystalline thin films.

For the crystalline structure, we used a rhombohedral unit cell described by the lattice parameters of 4.5 Å and 57.2° at 300 K based on experimental results.^[^ ^39^, ^40^ ^]^ The atomic positions in the unit cell were chosen as shown in 1d, where the distortion parameter τ was manually adjusted in the range between 0.00, corresponding to an undistorted structure, and 0.02.

For the DFT calculation, we employed a plane wave basis set with the PseudoDojo pseudo‐potential and the R2SCAN meta‐GGA exchange‐correlation functional,^[^ ^81^ ^]^ including spin‐orbit coupling. The energy cutoffs for the wave function and the density were set to 50 Ha and 200 Ha, respectively, while accurate ***k***‐point sampling was realized on a grid of 15 × 15 × 15 ***k***‐points.

The evaluation of the optical properties along a given path in ***k***‐space was achieved by applying the Kubo–Greenwood formula to each individual ***k***‐point of the band structure path.

### High‐Throughput Density of States Calculations

Density of states calculations have been performed again using the Quickstep code of CP2k. The TASK meta‐GGA description of the exchange‐correlation functional^[^ ^82^ ^]^ was employed as implemented in the libxc library.^[^ ^83^ ^]^ The functional combines an accurate description of the electronic structure around the bandgap (i.e., an exact estimation of the band gap)^[^ ^82^, ^84^ ^]^ and high efficiency, which allows for a large number of DOS calculations. The correlation part of the functional is given by a standard LDA description.^[^ ^85^ ^]^

## Conflict of Interest

The authors declare no competing interests.

## Supporting information

Supporting Information

## Data Availability

The data that support the findings of this study are available from the corresponding author upon reasonable request.
