# Supplementary material for: The Photoinduced Response of Antimony from Femtoseconds to Minutes
Source: Adv Mater. 2025 Jan 13;37(9):2414687. doi: 10.1002/adma.202414687 (PMC11881669; doi:10.1002/adma.202414687)
Supplement: Supplementary file 1 — Supporting Information [file ADMA-37-2414687-s001.pdf]

ADVANCED MATERIALS

Supporting Information

for *Adv. Mater.*, DOI 10.1002/adma.202414687

The Photoinduced Response of Antimony from Femtoseconds to Minutes

Sebastian Walfort, Nils Holle, Julia Vehndel, Daniel T. Yimam, Niklas Vollmar, Bart J. Kooi and Martin Salinga**

The Photoinduced Response of Antimony from Femtoseconds to Minutes - Supporting Information -

Sebastian Walfort Nils Holle Julia Vehndel Daniel T. Yimam Niklas Vollmar Bart J. Kooi Martin Salinga**

S. Walfort, N. Holle, J. Vehndel, N. Vollmar, Prof. Dr. M. Salinga
University of Münster, Institute of Materials Physics, Wilhelm-Klemm-Str. 10, 48149 Münster, Germany
Email Address: sebastian.walfort@uni-muenster.de, martin.salinga@uni-muenster.de
Dr. D. T. Yimam, Prof. Dr. B. J. Kooi
University of Groningen, Zernike Institute for Advanced Materials, Nijenborgh 3, 9747 Groningen, The Netherlands

Contents

1 Potential energy landscape of crystalline antimony **1**

2 Wavelet transform of coherent phonon oscillations **2**

3 Mapping distortions to excited electron densities **3**

4 Two-temperature model **3**

5 Optical properties **4**

6 Distortion motif in supercooled liquid antimony **5**

1 Potential energy landscape of crystalline antimony

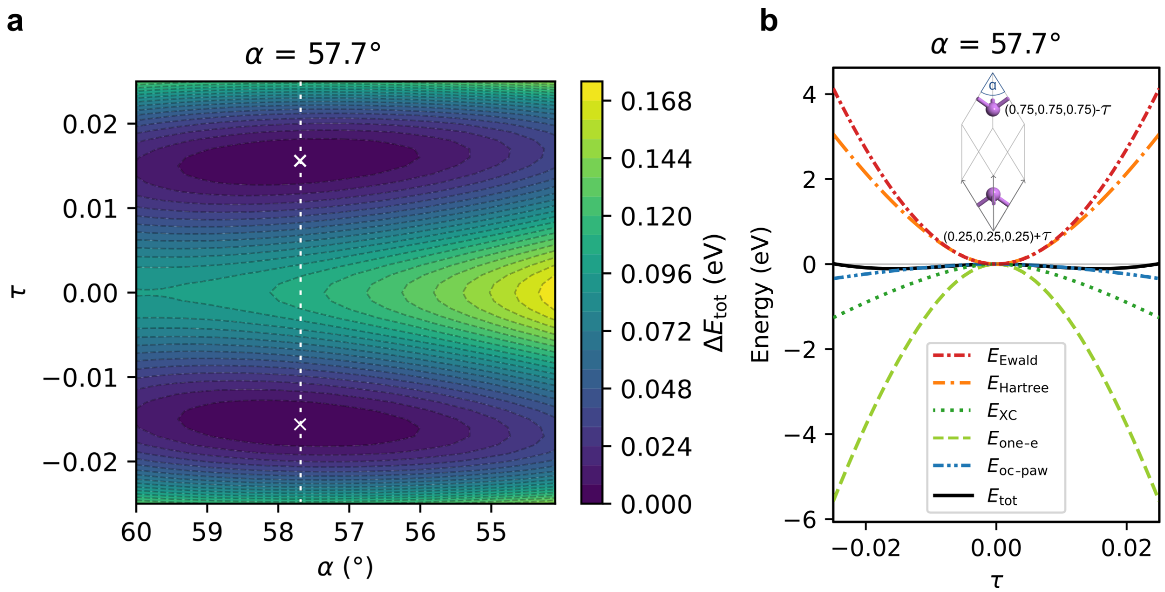

Figure S1: Contributions to the potential energy landscape. a) Potential energy landscape as a function of the two structural parameters α and τ of the rhombohedral unit cell of antimony. b) The different contributions to the potential energy for a constant angle $\alpha = 57.7°$ as a function of τ. The respective values at $\tau = 0$ are subtracted.

Following the approach by Argaman et al. for bismuth,[1] we calculate the different contributions to the potential energy landscape of crystalline antimony as a function of the two structural parameters α and τ. In Kohn-Sham Density Functional Theory (DFT), the total energy E_{tot} is defined as a function of the electronic density ρ, which is given by the sum of the following contributions[2]

$$E_{\text{tot}}(\rho) = E_{\text{one-e}}(\rho) + E_{\text{XC}}(\rho) + E_{\text{Hartree}}(\rho) + E_{\text{Ewald}} + E_{\text{oc-paw}}(\rho).$$

Here, $E_{\text{one-e}}$ represents the non-interacting kinetic energy, E_{XC} is the exchange-correlation energy, E_{Hartree} is the Hartree energy, which describes the Coulombic repulsion between the electrons, and E_{Ewald} describes the repulsion between the ions and $E_{\text{oc-paw}}$ is the one center contribution term from the projector augmented wave description.[3] While the two Coulombic repulsion terms drive the system towards lower distortion and ultimately to a simple cubic crystal structure with $\tau = 0$ and $\alpha = 60°$, the $E_{\text{one-e}}$ and E_{XC} terms push the system towards a distorted structure with lower symmetry. The sum of these opposing driving forces is a system with a shallow energy valley with a minimum at $\tau \approx 0.016$ (Figure S1b) that responds to a small perturbation with a large, directed structural change.

2 Wavelet transform of coherent phonon oscillations

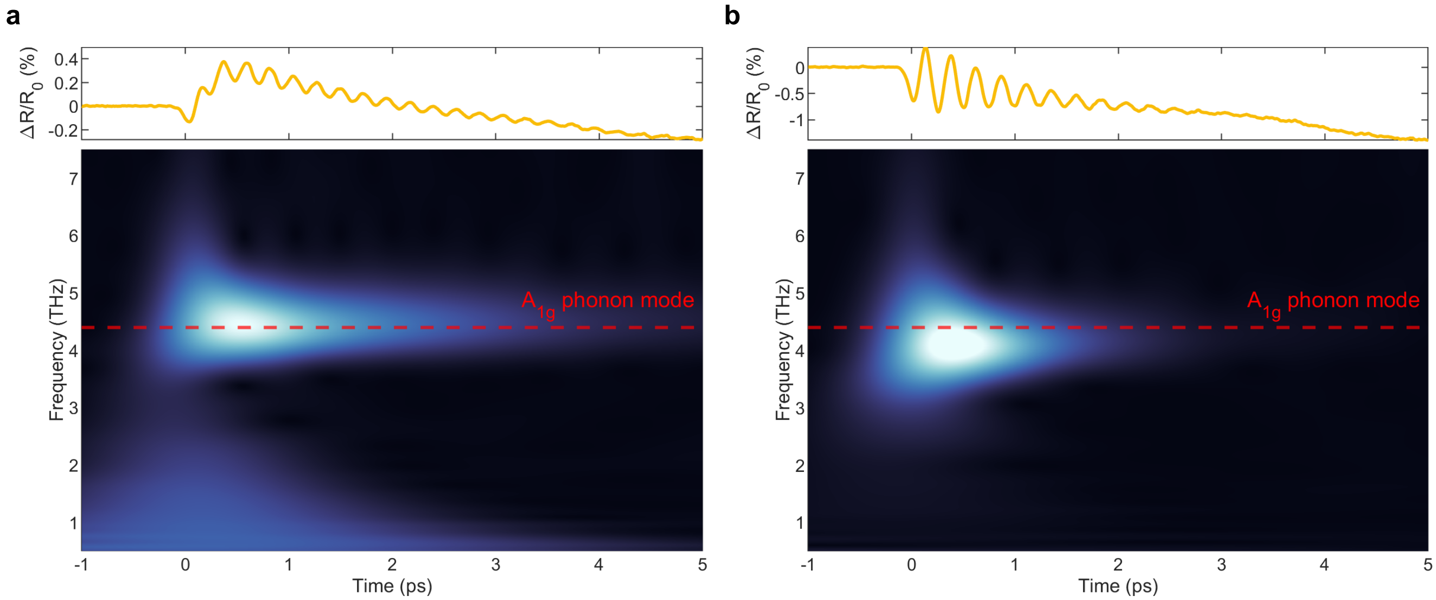

Figure S2: Continuous (Morlet) wavelet transform of exemplary measured change in reflectance for a low fluence excitation a) of 0.2% valence electrons and a medium fluence excitation b) of 0.7% valence electrons. Both measurements are on a 20 nm crystalline film at room temperature with a 730 nm probing wavelength. The higher fluence excitation is characterized by a small up-chirp in an initially softened phonon mode (compared to the low-fluence limit A_{1g} phonon mode in antimony) with an overall shorter lifetime.

3 Mapping distortions to excited electron densities

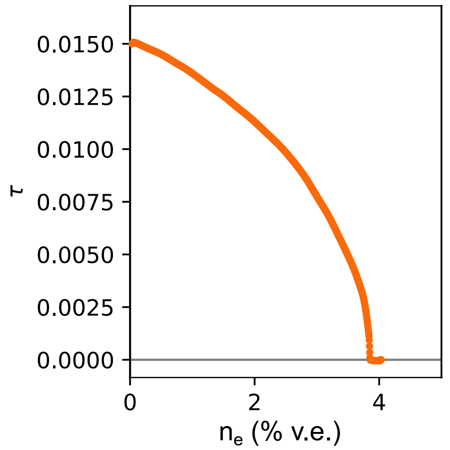

Figure S3: Dependence of quasi-equilibrium distortion τ on the excited electron density in percent valence electrons of antimony (data from O'Mahony[4]).

4 Two-temperature model

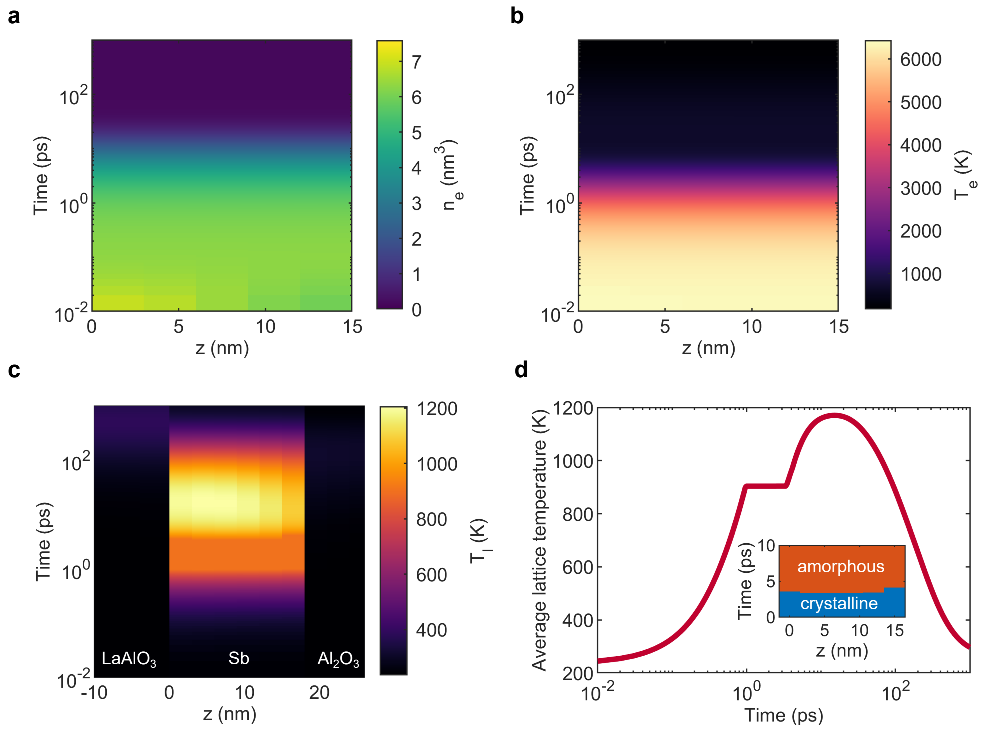

Figure S4: Results of the two temperature model. a) Temporal and spatial evolution of the excited electron density n_e in a 15 nm thick antimony film. Ambipolar diffusion quickly leads to a uniform distribution despite an initially exponential absorption profile. b) Temporal and spatial evolution of the electron temperature T_e. The uniform distribution in n_e and T_e leads to uniform heating of the antimony film. c) Temporal and spatial evolution of the lattice temperature T_l in the antimony film and the adjacent capping and substrate. Upon reaching the melting temperature of antimony, the temperature plateaus and only continuous to increase once the melting enthalpy is reached. Cooling is limited by the thermal interface conductances to capping and substrate. As a consequence, the temperature within the antimony film remains uniform and the temperature increase in capping and substrate is negligible. d) Corresponding averaged temperature of the antimony film as a function of time. The inset shows the phase of the antimony film during the first 10 ps.

5 Optical properties

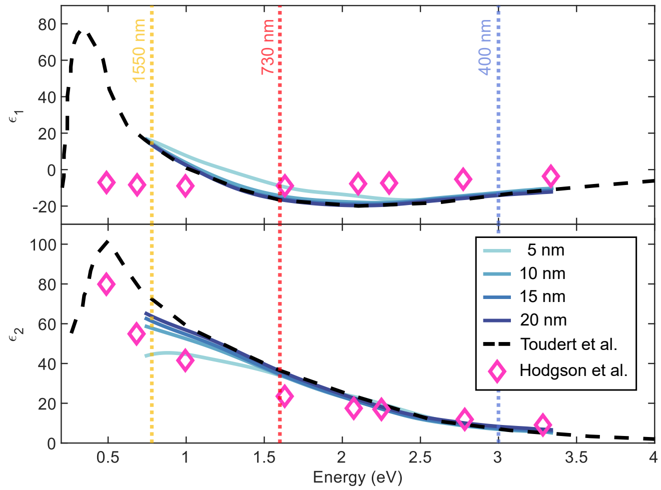

Figure S5: Measured optical properties of antimony. Real and imaginary part of the permittivity for four different crystalline antimony film thicknesses. The measured permittivities agree very well with literature data for bulk antimony (from Toudert et al.[5]) except for the thinnest sample. Quantisation effects that eventually lead to the further opening of the bandgap in few bi-layer antimony probably might already affect the 5 nm film and lead to a reduction in interband absorption. Also shown are the positions of the three probing wavelengths of the switching experiments and experimental data for liquid antimony (from Hodgson et al.[6]).

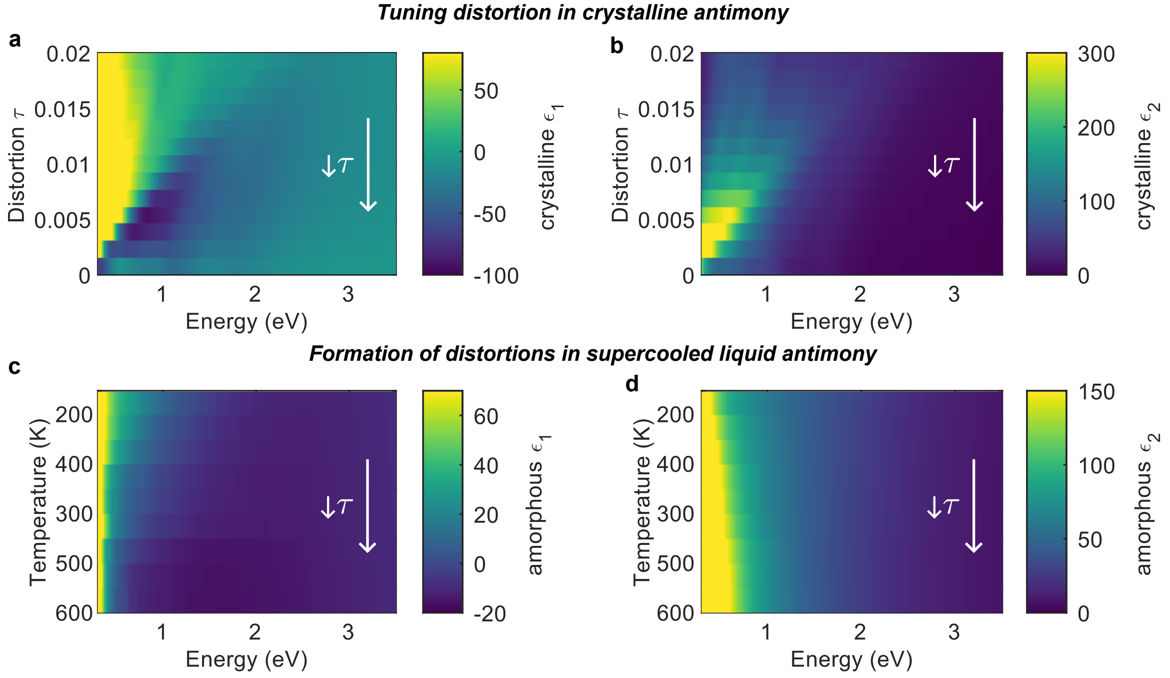

Figure S6: Role of Peierls-like distortions for the optical properties of crystalline and supercooled liquid / vitreous antimony. a), b) Calculated real and imaginary permittivity for crystalline antimony for different static distortions τ. The main manuscript (Figure 2a) compares the experimentally determined and calculated change in permittivity at a wavelength of 730 nm (1.7 eV). The broader energy range plotted here illustrates the overall blue (red) shift of interband transitions with increasing (decreasing) distortion with the accompanying opening (closing) of the electronic band gap. c), d) Calculated real and imaginary permittivity of supercooled liquid / vitreous antimony during quenching in molecular dynamics simulations. As discussed in the main manuscript (around Figure 3b), a Peierls-like distortion motif emerges and continuously increases in amplitude with decreasing temperature, which in turn is accompanied by the opening of a pseudo-gap. The optical transitions consequently undergo a blue (red) shift with decreasing (increasing) temperatures.

6 Distortion motif in supercooled liquid antimony

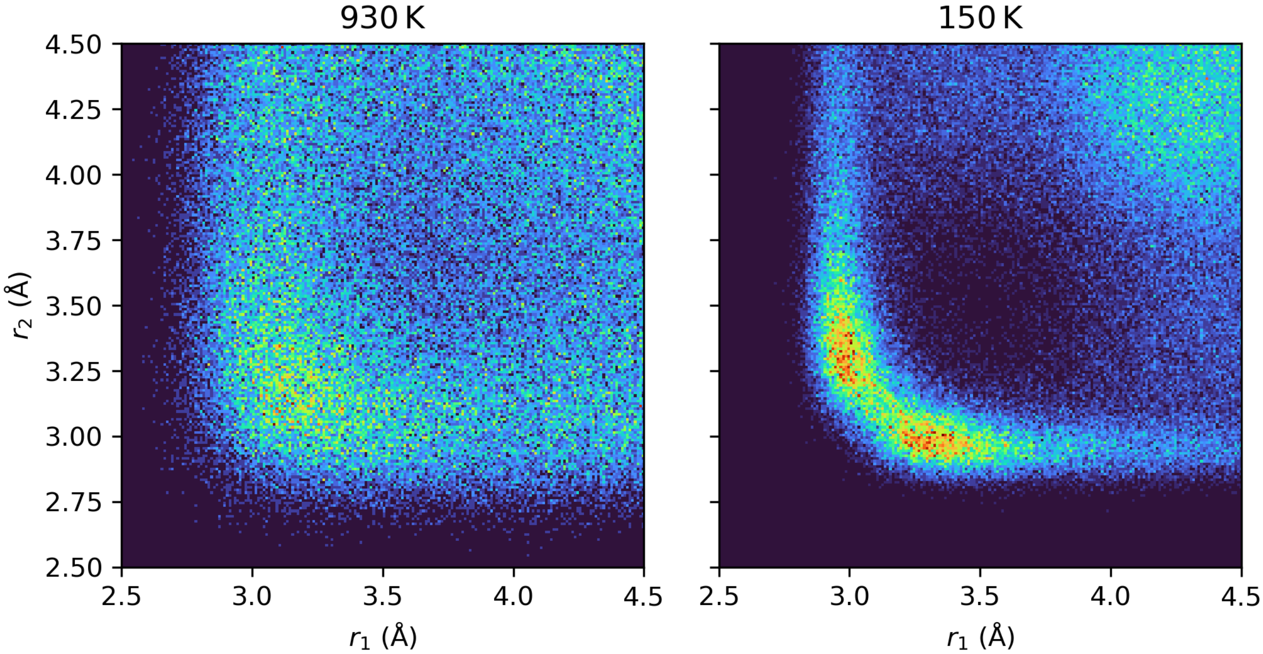

Figure S7: Distortion in amorphous antimony, comparing the Peierls-like distortion motif in high temperature liquid antimony and in the low temperature glass through the angular-limited three-body correlation (ALTBC) function.

The Angular-limited three-body correlation (ALTBC) is a higher-order correlation function that is defined as

$$g^{(3)}(r, r') = \frac{V}{\rho(N-1)(N-2)} \sum_{i_1,i_2,i_3} \langle \delta(r - r_{12}) \times \delta(r' - r_{23}) \Theta(\beta - \delta) \rangle,$$

with i_1, i_2 and i_3 distinct indices of atoms, V the unit cell volume, N the number of particles, $\rho = V/N$ the atomic density,

$$\cos\beta = \frac{\mathbf{r}_{i_1 i_2} \cdot \mathbf{r}_{i_2 i_3}}{r_{12} r_{23}}$$

the alignment angle and δ an angular threshold, typically 25° in phase change materials.[7]

References

[1] U. Argaman, D. Kartoon, G. Makov, *J. Phys. Condens. Matter* **2019**, *31*, 46 465501.

[2] R. G. Parr, Y. Weitao, Oxford University Press, ISBN 9780195092769, **1995**.

[3] P. E. Blöchl, *Phys. Rev. B* **1994**, *50*, 24 17953.

[4] S. M. O'Mahony, Ph.D. thesis, University College Cork, **2020**.

[5] J. Toudert, R. Serna, *Opt. Mater. Express* **2016**, *6*, 7 2434.

[6] J. N. Hodgson, *Philos. Mag.-J. Theor. Exp. Appl. Phys.* **1962**, *7*, 74 229.

[7] I. Ronneberger, Dissertation, RWTH Aachen University, Aachen, **2016**.
